# The Existence of at Least Three Genomic Signature Patterns and at Least Seven Subtypes of COVID-19 and the End of the Disease

**DOI:** 10.3390/vaccines10050761

**Published:** 2022-05-11

**Authors:** Zhengjun Zhang

**Affiliations:** Department of Statistics, University of Wisconsin, Madison, WI 53706, USA; zjz@stat.wisc.edu; Tel.: +1-(608)-262-2598

**Keywords:** direct gene effects, indirect gene effects, COVID-19 detection, gene-gene interaction, competing risks

## Abstract

Hoping to find genomic clues linked to COVID-19 and end the pandemic has driven scientists’ tremendous efforts to try all kinds of research. Signs of progress have been achieved but are still limited. This paper intends to prove the existence of at least three genomic signature patterns and at least seven subtypes of COVID-19 driven by five critical genes (the smallest subset of genes) using three blood-sampled datasets. These signatures and subtypes provide crucial genomic information in COVID-19 diagnosis (including ICU patients), research focuses, and treatment methods. Unlike existing approaches focused on gene fold-changes and pathways, gene-gene nonlinear and competing interactions are the driving forces in finding the signature patterns and subtypes. Furthermore, the method leads to high accuracy with hospitalized patients, showing biological and mathematical equivalences between COVID-19 status and the signature patterns and a methodological advantage over other methods that cannot lead to high accuracy. As a result, as new biomarkers, the new findings and genomic clues can be much more informative than other findings for interpreting biological mechanisms, developing the second (third) generation of vaccines, antiviral drugs, and treatment methods, and eventually bringing new hopes of an end to the pandemic.

## 1. Introduction

Since the virus SARS-CoV-2 was first reported in December 2019, numerous research results related to the virus and COVID-19 disease have been published. Scientists have put tremendous effort into trying to find genomic clues linked to COVID-19. However, knowledge of COVID-19 is still limited, and many problems have remained unanswered [1,2,3,4,5,6,7,8,9,10,11,12]. As a result, many published results have not guaranteed convincing accuracy. With an exception, a data science study by Zhang (2021) reported five critical genes, and their combined effects can accurately classify COVID-19 patients and COVID-19 free patients into their respective groups and further classify COVID-19 patients into seven subtypes [2].

The analytical method used in this study has been proven a powerful approach in earlier studies where breast cancer patients, colorectal cancer patients, and lung cancer patients are again classified into their respective groups (nearly) without errors among eleven different study cohorts with thousands of patients [2,13,14,15]. For example, in a breast cancer study, eight widely known genes—BRCA1, BRCA2, PALB2, BARD1, RAD51C, RAD51D, ATM—were proved to have low efficacy in terms of diagnosis [13]. In addition, a colorectal cancer study showed CXCL8 alone could predict early-stage colorectal cancer accurately [14]. It was surprising that such a unique feature has been missed in the literature using other existing analytical methods.

This paper intends to prove the existence of at least three genomic signature patterns and at least seven subtypes of COVID-19 driven by five critical genes (the smallest subset of genes). For this purpose, we are going to advance further the signature patterns found in the earlier study by Zhang (2021) using a different dataset generated by the same study in Overmyer et al. (2020) [1]. This paper is completely new in its conceptual framework in biological and mathematical equivalence compared with earlier pure data analysis. This new study conducted a competing risk analysis using the max-linear logistic regression model to analyze 126 blood samples from COVID-19-positive and COVID-19-negative patients [1]. The two sampled groups are: lab-confirmed COVID-19 hospitalized patients and the control is other disease types of hospitalized patients, including ICU cases. There are two types of datasets available. One type is TPM (transcripts per million), while another type is EC (expected counts), which are analyzed in this paper. Both datasets led to competing COVID-19 risk classifiers derived from 19,472 genes and their differential expression values. The final classifier models for both datasets only involve five critical genes, ABCB6, KIAA1614, MND1, SMG1, and RIPK3, which led to 100% sensitivity and 100% specificity of the 126 samples. The two datasets naturally cross-validate the efficiency of the discovered five genes. These five genes also form signature patterns as new biomarkers and classify COVID-19 patients into seven subgroups coded by involved genes, sub-I (KIAA1614, MND1, SMG1), sub-II (ABCB6, MND1, SMG1), sub-III (RIPK3), sub-IV (ABCB6, KIAA1614, MND1, SMG1), sub-V (KIAA1614, MND1, RIPK3, SMG1), sub-VI (ABCB6, MND1, RIPK3, SMG1), sub-VII (ABCB6, KIAA1614, MND1, SMG1, RIPK3), and heterogeneous populations. We note that the control group was hospitalized patients, including non-ICU patients and ICU patients [1]. As a result, the genes identified can be classified as COVID-19 specific. Given their high accuracy in predicting COVID-19-positive or -negative status, these five genes can be critical in developing proper, focused, and accurate COVID-19 testing procedures, guiding the second-generation vaccine development, studying antiviral drugs, and treatments. Furthermore, the accurate results prove the biological equivalences between COVID-19 status and the signature patterns and mathematically establish the correspondences. Such a phenomenon is fundamentally meaningful for conducting further focused research on these five genes and other highly correlated genes (e.g., DBN1, LY6G6C, TMEM54, MTMR1, SNORC, ANP32E, ATAD2, SMC2, ZWILCH, SMC4, C6orf47, STRADA, LRSAM1, UNC93B1, SASH3) to these five, leading to the second (third) generation of vaccines, antiviral drugs, and treatment methods.

We further test the above five critical genes using a third dataset published in *Science* by Arunachalam et al. [16]. The dataset contains 64083 genes and 17 COVID-19 subjects and 17 healthy controls. We found that four genes, ABCB6, KIAA1614, MND1, and SMG1, can accurately predict all subject labels.

Among these five genes, KIAA1614 is an uncharacterized protein gene, according to genecards.org. This gene could be fundamental. SMG1 has the description: nonsense mediated mRNA decay associated PI3K related kinase. It could be essential for developing second-generation mRNA vaccines. According to the literature, nonsense mediated mRNA decay plays a decisive role in monitoring and controlling protein changes. The mRNA gene SMG1 showed two different signs (+, −) in two formulas and did not appear in the third formula presented in Section 3, which suggests that we need three different types (both mRNA and non-mRNA) of vaccines to cover the entire possible spectrum of COVID-19 variants. Our proven classifiers show that one particular vaccine may only be effective for one type of virus caused by one signature pattern and one subtype among three signature patterns (CF-I, CF-II, and CF-III in Section 3.3) and seven subtypes, which can explain the high percentages of breakthrough infections; see Section 3.4 and Section 3.5 for detailed explanations.

The rest of the paper is organized as follows. In Section 2, the methodology is briefly summarized. Then, Section 3 presents the data, the derived competing classifiers, and the interpretations. Finally, in Section 4, conclusions and discussions on the findings, future directions, and limitations are discussed.

## 2. Methodology

This methodological section briefly introduces max-linear competing factor classifiers for self-contained due to different data structures used in this work compared with other research, whose details for data structures can be found in the papers [2,13,14,15].

Suppose $Y_{i}$ is the ith individual patient’s COVID-19 status ($Y_{i}\;=\;0$ as not infected, $Y_{i}\;=\;1$ for infected) and $X_{i}^{\left(k\right)}\;=\;\left(X_{i1}^{\left(k\right)},X_{i2}^{\left(k\right)},\ldots ,X_{ip}^{\left(k\right)}\right),k\;=\;1,\ldots ,K,$ are the gene expression values with p = 19,472 genes in this study. Here k stands for the kth type of gene expression level drawn based on K different biological sampling methodologies. Note that most published works set K = 1, and hence, the superscript $\left(k\right)$ can be dropped from the predictors. In this paper, K = 2 (TPM and EC). Using a logit link (or other monotone links), we can model the risk probability $p_{i}^{\left(k\right)}$ of the ith person’s infection status as:

(1) $$\log\left(\frac{p_{i}^{\left(k\right)}}{1-p_{i}^{\left(k\right)}}\right)\;=\;\beta_{0}^{\left(k\right)}\;+\;X_{i}^{\left(k\right)}\beta^{\left(k\right)}$$

alternatively, we write

$$p_{i}^{\left(k\right)}\;=\;\frac{\exp\left(\beta_{0}^{\left(k\right)}\;+\;X_{i}^{\left(k\right)}\beta^{\left(k\right)}\right)}{1\;+\;\exp\left(\beta_{0}^{\left(k\right)}\;+\;X_{i}^{\left(k\right)}\beta^{\left(k\right)}\right)}$$

where $\beta_{0}^{\left(k\right)}$ is an intercept, $X_{i}^{\left(k\right)}$ is a 1 × p observed vector, and $\beta^{\left(k\right)}$ is a p × 1 coefficient vector which characterizes the contribution of each predictor (gene in this study) to the risk.

Considering there have been several variants of SARS-CoV-2 and multiple symptoms (subtypes) of COVID-19 diseases, it is natural to assume that the genomic structures of all subtypes could be different. Suppose that all subtypes of COVID-19 diseases may be related to G groups of genes

(2) $$\Phi_{ij}^{\left(k\right)}\;=\;\left(X_{i,j_{1}}^{\left(k\right)},X_{i,j_{2}}^{\left(k\right)},\ldots ,X_{i,j_{g_{j}}}^{\left(k\right)}\right),j\;=\;1,\ldots ,G,g_{j}\geq 0,k\;=\;1,\ldots ,K$$

where i is the ith individual in the sample, and $g_{j}$ is the number of genes in jth group. Note that we do not use the widely used gene pathways in our newly developed machine learning algorithm. It is possible that blind pursuit of gene pathways may lead to wrong directions and lose chances of finding the scientific truth. Instead, our methodological approach will automatically find the smallest numbers of G and $g_{j}$ to reach 100% accuracy, and as a result, better interpretations can be achieved.

The competing (risk) factor classifier is defined as

(3) $$\log\left(\frac{p_{i}^{\left(k\right)}}{1-p_{i}^{\left(k\right)}}\right)\;=\;max\left(\beta_{01}^{\left(k\right)}\;+\;\Phi_{i1}^{\left(k\right)}\beta_{1}^{\left(k\right)},\beta_{02}^{\left(k\right)}\;+\;\Phi_{i2}^{\left(k\right)}\beta_{2}^{\left(k\right)},\ldots ,\beta_{0G}^{\left(k\right)}\;+\;\Phi_{iG}^{\left(k\right)}\beta_{G}\right)\;$$

where $\beta_{0j}^{\left(k\right)}$’s are intercepts, $\Phi_{ij}^{\left(k\right)}$ is a $1\;\times \;g_{j}$ observed vector, $\beta_{j}^{\left(k\right)}$ is a $g_{j}\;\times \;1$ coefficient vector which characterizes the contribution of each predictor in the j group to the risk.

**Remark** **1.** *In (3),* $p_{i}^{\left(k\right)}$ *is mainly related to the largest component* $\beta_{0j}^{\left(k\right)}\;+\;\Phi_{ij}^{\left(k\right)}\beta_{j}^{\left(k\right)},j\;=\;1,\ldots ,G$ *, i.e., all components compete to take the most significant effect.*

**Remark** **2.** *Taking* $\beta_{0j}^{\left(k\right)}\;=\;-\infty ,j\;=\;2,\ldots ,G$ *, (3) is reduced to the classical logistic regression, i.e., the classical logistic regression is a special case of the new classifier. Compared with black-box machine learning methods (e.g., random forest, deep learning (convolutional) neural networks (DNN, CNN)) and regression tree methods, each competing risk factor in (3) forms a clear, explicit, and interpretable signature with the selected genes. The number of factors corresponds to the number of signatures, i.e.,* G *. This model can be a bridge between linear models and more advanced machine learning methods (black box) models. However, (3) remains the desired properties of interpretability, computability, predictability, and stability. Note that this remark is similar to Remark 1 in Zhang (2021) [15].*

In practice, we have to choose a threshold probability value to decide a patient’s class label. Following the general trend in the literature, we set the threshold to be 0.5. As such, if $p_{i}^{\left(k\right)}\leq 0.5$, the ith individual is classified as disease-free; otherwise, the individual is classified to have the disease.

With the above-established notations, we introduce a new machine learning classifier, smallest subset and smallest number of signatures (S4), for K = 1 to K = 2 as follows.

(4) $$\left(\hat{\beta },\hat{S},\hat{G}\right)\;=\;{\mathrm{argmin}}_{\beta ,S_{j}\subset S,j\;=\;1,2,\ldots ,G}\{{\left(1\;+\;\lambda_{1}\;+\;\left|S_{u}\right|\right)}^{\sum_{1\leq k\leq K;1\leq i\leq n}(I\left(p_{i}^{\left(k\right)}\leq 0.5\right)I\left(Y_{i}\;=\;1\right)\;+\;I(p_{i}^{\left(k\right)}>0.5)I\left(Y_{i}\;=\;0\right))}\;+\;\lambda_{2}\left(\left|S_{u}\right|-\frac{\left|S_{u}\right|\;+\;G-1}{\left(\left|S_{u}\right|\;+\;1\right)\;\times \;G-1}\right)\}$$

where $I\left(.\right)$ is an indicative function, $p_{i}^{\left(k\right)}$ is defined in Equation (3), $S\;=\;\left\{1,2,\ldots ,19472\right\}$ is the index set of all genes, $S_{j}\;=\;\left\{j_{j1},\ldots ,j_{j,g_{j}}\right\}$, j = 1,…,G are index sets corresponding to (2), $S_{u}$ is the union of $\left\{S_{j},j\;=\;1,\ldots ,G\right\}$, $\left|S_{u}\right|$ is the number of elements in $S_{u}$, $\lambda_{1}\geq 0$ and $\lambda_{2}\geq 0$ are penalty parameters, and $\hat{S}\;=\;\left\{j_{j1},\ldots ,j_{j,g_{j}},j\;=\;1,\ldots ,\hat{G}\right\}$ and $\hat{G}$ are the final gene set selected in the final classifiers and the number of final signatures.

**Remark** **3.** *The case of* K = 1 *corresponds to the classifier introduced in Zhang (2021) [15]. The case of* K = 1 *and* $\lambda_{2}\;=\;0$ *corresponds to the classifier introduced in Zhang (2021) [2].*

The following Proposition 1 mathematically proves the existence of desired solutions. The proof follows the lines in the work by extending K = 1 to K = 2 [15].

**Proposition** **1.** *Suppose the smallest number that* $\Delta \;=\;\sum_{1\leq k\leq K;1\leq i\leq n}(I\left(p_{i}^{\left(k\right)}\leq 0.5\right)I\left(Y_{i}\;=\;1\right)\;+\;I(p_{i}^{\left(k\right)}>0.5)I\left(Y_{i}\;=\;0\right))$ *can reach is* m *. Then, for suitable choices of* $\lambda_{1}\geq 0$ *with* $\lambda_{1}\;+\;\left|S_{u}\right|>0$ *and* $\lambda_{2}\geq 0$ *, the new classifier S4 will lead to the smallest* $\left|S_{u}\right|$ *and the smallest number of* G *such that* Δ = m.

**Remark** **4.** *A perfect classifier (100% sensitivity and 100% specificity) will have* m = 0 *in Equation (4), which is the case in our study. We note that only with* m = 0 *and the smallest subset of genes, the mathematical and biological equivalence between the disease and the selected genes can be established.*

## 3. Data Descriptions, Results, and Interpretations

### 3.1. The First and Second Datasets

The COVID-19 data to be analyzed is publicly available as GSE157103: large-scale multi-omic analysis of COVID-19 severity, public on 29 August 2020 [1]. The experiment type is “Expression profiling by high throughput sequencing”. One hundred and twenty-six samples were analyzed in total, with 100 COVID-19 hospitalized patients and 26 hospitalized non-COVID-19 patients. There are two types of datasets available. One type is TPM (transcripts per million), while another type is EC (expected counts). The prior analysis outcome of TPM data was reported in Zhang (2021) [2]. This new study targets EC data and makes comparisons to TPM data. We note that among 100 COVID-19 patients, 50 are ICU patients and 50 are non-ICU hospitalized patients. Among 26 COVID-19 free patients, 16 of them are ICU patients with other types (non-COVID-19) of disease, and 10 of them are non-ICU patients with other types of disease.

### 3.2. The Third Dataset

The third set of COVID-19 data to be analyzed is publicly available as GSE152418: RNAseq analysis of PBMCs in a group of 17 COVID-19 subjects and 17 healthy controls, public on 13 June 2020 and last updated on 20 May 2021 [16]. Illumina bcl2fastq v2.17.1.14 was used for demultiplexing. Reads were mapped to the human (GRCh38 Ensemble release 100) genomic reference with STAR (v2.7.3a) with default alignment parameters. Abundance estimation of raw read counts per transcript was done internally with STAR using the algorithm of htseq-count.

In the following subsections, we first focus on the first two datasets: TPM and EC.

### 3.3. The Competing Factor Classifiers and Their Resulting Risk Probabilities

Solving the optimization problem (4) among 19,472 genes with K = 2 (k = 1 for TPM data, and k = 0 for EC data) using the Monte Carlo search method, we obtain the following classifiers with five critical genes (ABCB6 (ATP binding cassette subfamily B member 6—Langereis blood group), KIAA1614 (uncharacterized protein), MND1 (meiotic nuclear divisions 1), SMG1 (nonsense mediated mRNA decay associated PI3K related kinase), RIPK3 (receptor interacting serine/threonine kinase 3)):

(5) $$\begin{array}{l} \mathrm{CF}-I\;(\mathrm{TPM}):-0.3303\;+\;3.4153\;\times \;\mathrm{KIAA}1614-0.1248\;\times \;\mathrm{SMG}1\;+\;0.2177\;\times \;\mathrm{MND}1\\ \mathrm{CF}-\mathrm{II}\;(\mathrm{TPM}):-0.7378-0.4620\;\times \;\mathrm{ABCB}6\;+\;0.0654\;\times \;\mathrm{SMG}1\;+\;0.9093\;\times \;\mathrm{MND}1\;\\ \mathrm{CF}-\mathrm{III}\;(\mathrm{TPM}):\;\;\;\;\;6.9283-0.3921\;\times \;\mathrm{RIPK}3\\ \mathrm{CF}\;\left(\mathrm{TPM}\right)\;=\;\max\{\mathrm{CF}-I\left(\mathrm{TPM}\right),\;\mathrm{CF}-\mathrm{II}\left(\mathrm{TPM}\right),\;\mathrm{CF}-\mathrm{III}\left(\mathrm{TPM}\right)\} \end{array}$$

(6) $$\begin{array}{l} \mathrm{CF}-I\;(\mathrm{EC}):-0.7877\;+\;0.0351\;\times \;\mathrm{KIAA}1614-0.0008\;\times \;\mathrm{SMG}1\;+\;0.0181\;\times \;\mathrm{MND}1\\ \mathrm{CF}-\mathrm{II}\;(\mathrm{EC}):-4.6701-0.0408\;\times \;\mathrm{ABCB}6\;+\;0.0014\;\times \;\mathrm{SMG}1\;+\;0.2134\;\times \;\mathrm{MND}1\;\\ \mathrm{CF}-\mathrm{III}\;(\mathrm{EC}):\;\;\;\;\;3.1584-0.0042\;\times \;\mathrm{RIPK}3\\ \mathrm{CF}\;(\mathrm{EC})\;=\;\max\{\mathrm{CF}-I(\mathrm{EC}),\;\mathrm{CF}-\mathrm{II}(\mathrm{EC}),\;\mathrm{CF}-\mathrm{III}(\mathrm{EC})\} \end{array}$$

where Equation (5) is for TPM data which were first reported in Zhang (2021) [2], while Equation (6) is for the EC data. In all equations, (TPM) stands for data being TPM, and (EC) means data are expected counts. In Equation (5), CF-I (TPM) is the first component classifier derived from TPM data and three critical genes, KIAA1614, SMG1, and MND1. CF-II (TPM) is the second component classifier derived from TPM data and three critical genes, ABCB6, SMG1, and MND1. CF-III (TPM) is the third component classifier derived from TPM data using one gene, RIPK3 alone. CF (TPM) is the final combined classifier with competing component classifiers (signatures) of CF-I (TPM), CF-II (TPM), and CF-III (TPM). Other competing classifiers for EC are similarly defined.

The risk probabilities of each of three component classifiers based on TPM are

(7) $$P-i-(\mathrm{TPM})\;=\;\frac{\exp\left(\mathrm{CF}-i-(\mathrm{TPM})\right)}{1\;+\;\exp\left(\mathrm{CF}-i-(\mathrm{TPM})\right)},i\;=\;I,\;\mathrm{II},\;\mathrm{III}\;$$

and the risk probabilities based on all three component classifiers together are

(8) $$P-(\mathrm{TPM})\;=\;\frac{\exp\left(\mathrm{CF}(\mathrm{TPM})\right)}{1\;+\;\exp\left(\mathrm{CF}(\mathrm{TPM})\right)}.\;$$

Similarly, the risk probabilities calculated from EC are

(9) $$P-i-(\mathrm{EC})\;=\;\frac{\exp\left(\mathrm{CF}-i-(\mathrm{EC})\right)}{1\;+\;\exp\left(\mathrm{CF}-i-(\mathrm{EC})\right)},i\;=\;I,\;\mathrm{II},\;\mathrm{III}\;$$

and the risk probabilities based on all three component classifiers together are

(10) $$P-(\mathrm{EC})\;=\;\frac{\exp\left(\mathrm{CF}(\mathrm{EC})\right)}{1\;+\;\exp\left(\mathrm{CF}(\mathrm{EC})\right)}.\;$$

Table 1 presents the performance (accuracy, sensitivity, specificity) of each individual classifier in Equations (5) and (6).

From Table 1, we can see that the coefficients of MND1 are different between CF-I and CF-II, and the signs of SMG1 are different. This phenomenon tells us that the function of each gene and its contribution to the risk probability depends on other factors (genes) in the combination, i.e., there are gene-gene interactions and gene-subtype interactions. We note that such interactions are hardly expressed in existing models, and as a result, the interpretation of other types of current models can be difficult. We also notice that the sensitivities of individual classifiers are low, and at first glance, we may think such models are not powerful enough to be used in practice. Given the combined classifiers’ superior performance, we can immediately infer that the populations are heterogeneous, and as a result, a single model in the literature must have low performance in all samples. A combination of individual classifiers should lead to better performance. However, the number of genes can be large, and their practical value can be in doubt. Fortunately, our S4 classifiers will not suffer such problems.

Table 2 and Table 3 present some patients’ gene expression values, competing classifier factors, and predicted probabilities defined in Equations (5)–(10). For full tables, we refer to tables in a Supplementary Excel File.

**Figure 1:**
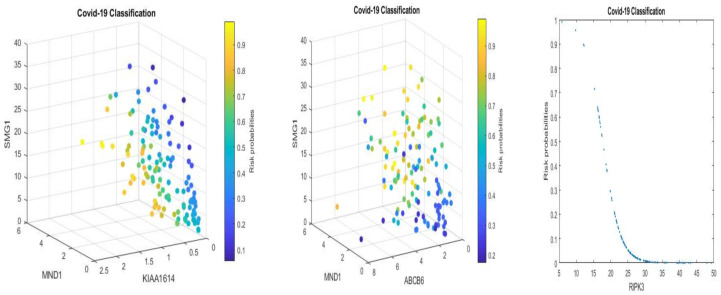
COVID-19 classifiers using TPM data with axis units corresponding to Table 2: visualization of gene-gene relationship and gene-risk probabilities. Note that 0.5 is the probability threshold. The left panel is for CF-I with the X-axis being MND1, Y-axis being KIAA1614, and Z-axis being SMG1. The risk probability bar is on the right. The middle panel is for CF-II with the X-axis being MND1, Y-axis being ABCB6, and Z-axis being SMG1. The risk probability bar is on the right. The right panel is for CF-III with the X-axis being RIPK3 and the Y-axis being the risk probability.

**Figure 2:**
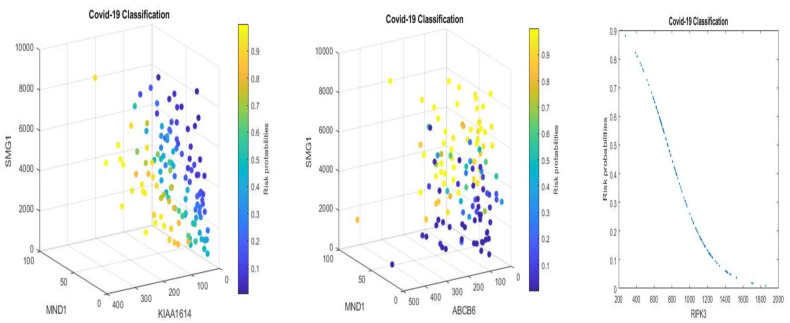
COVID-19 classifiers using EC data with the units corresponding to Table 3: visualization of gene-gene relationship and gene-risk probabilities. Note that 0.5 is the probability threshold. The left panel is for CF-I with the X-axis being MND1, Y-axis being KIAA1614, and Z-axis being SMG1. The risk probability bar is on the right. The middle panel is for CF-II with the X-axis being MND1, Y-axis being ABCB6, and Z-axis being SMG1. The risk probability bar is on the right. The right panel is for CF-III with the X-axis being RIPK3 and the Y-axis being the risk probability.

Figure 1 and Figure 2 present critical gene expression levels and risk probabilities corresponding to different measurement scales (TPM and EC) and different component competing factor classifiers in Table 2 and Table 3.

From Figure 1 and Figure 2, we can see clear patterns of how the patients are classified and how they are correlated with individual genes. For example, some patients can be classified by one gene, RIPK3 (the right panels in the figures), while some patients are classified by the combined effects of linear combinations of three genes (the left and middle panels). As a result, these observations justify the existence of three genomic signature patterns, i.e., the three competing classifiers of COVID-19.

We can also see similar patterns between Figure 1 and Figure 2. This phenomenon is mainly due to the component genes and signs of coefficients in the classifiers CF-i-(TPM) and CF-i-(EC) being the same. In addition, the linear correlation coefficients between TMP and EC data for genes ABCB6, KIAA1614, MND1, RIPK3, and SMG1, are 0.87, 0.94, 0.95, 0.68, and 0.93, respectively, which supports the pattern similarity.

### 3.4. The Combination Effects and the Competing Factor Effects

The same signs of coefficients in the classifiers CF-i-(TPM) and CF-i-(EC) reveal that these classifiers are robust to nonlinear transformations and the five genes are critical and COVID-19 specific (recall that the patients in the control group are also hospitalized patients with 16 of them being ICU patients).

The pairwise correlation coefficients among these five genes are presented in Table 4. The table shows that TPM data and EC data show different gene-gene correlations. Even so, they still lead to the same accuracy. As a result, they can be used as cross-validation of the proposed S4 model (4) and the selected genes. Finally, we note that the classical cross-validation works under a homogeneous population, i.e., it does not apply to heterogeneous populations.

From Equations (5) and (6), we can see that increasing ABCB6 and RIPK3 expression levels (TPM) and decreasing KIAA1614 and MND1 expression levels will help the patients. However, from Table 4, we see that ABCB6 is positively correlated with KIAA1614 and MND1, and then, an increase in ABCB6 expression level may result in an increase in MND1 expression level and KIAA1614 expression level, which increases the COVID-19 competing risk CF-I. As a result, any efficient treatments of COVID-19 must consider the functional effects of the discovered genes.

Note that RIPK3 does not appear in the classifiers CF-I and CF-II, and the signs of SMG1 in CF-I and CF-II are different. As a result, a vaccine/antiviral drug/therapy which is based on the function of SMG1 (an mRNA gene, with positive and negative coefficient signs) in the CF-I (CF-II) may benefit the patients classified in the CF-I (CF-II). However, it may not have any effects on the patients in the CF-III. Furthermore, it may make the status of patients in the CF-II (CF-I) worse. Analogously, a vaccine/antiviral drug/therapy which is based on the function of RIPK3 (CF-III) may not be effective for the patients affected by the effects of CF-I and CF-II. The vaccines based on the current knowledge and technology have not used any messages from gene-gene interactions and gene-subtype interactions. Given the uncertainty and the unknown side effects of the first-generation vaccines, if the next generation vaccines can utilize the information discovered in the genomic signature patterns of COVID-19, it can be hoped the efficacy can be largely improved, especially for those mRNA type vaccines. As such, these observations reveal that we may need at least three different types of vaccines against COVID-19 subtypes (variants).

Note that these five critical genes have not been reported in any papers except Zhang (2021) [2]. They are not from any single gene pathway. Analogously, their functions may be described as a basketball team’s combination effects. First, in a basketball team, there are five positions: center (C), power forward (PF), small forward (SF), point guard (PG), and shooting guard (SG). A combination of ABCB6-MND1-SMG1 (KIAA1614-MND1-SMG1) may be described as a driving force of a powerful PF-C-PG (SF-C-PG) combination in scoring, and RIPK3 is like a powerful SG. Second, the expression levels are comparable to the playing time of the players and their roles in the rotations competing against different opponents and their playing combinations. Third, the driving forces of winning games can be either one or two or all of the three combinations.

Note that the correlation coefficients among the five genes calculated from TPM (upper triangle) and EC (lower triangle) in Table 4 are different. This phenomenon can be explained by the nonlinear relationship between TPM and EC, within TPM and EC, and heterogeneous populations among patients, which is a perfect scenario for the proposed model in Section 2. In addition, note that due to 100% accuracy, metrics such as ROC, recall, and precision are also with 100% accuracy.

### 3.5. The Existence of Subtypes

In Section 3.3, we saw that each signature could be used as a classifier given its 100% specificity. However, from Figure 1 and Figure 2, we see that some patients can only be classified by one particular signature classifier, some patients can only be classified by two competing classifiers, and some patients can be classified by any of the three competing classifiers. This observation shows that COVID-19 patients are heterogeneous, and their COVID-19 status can be further classified into subtypes using the classifier combinations.

**Figure 3:**
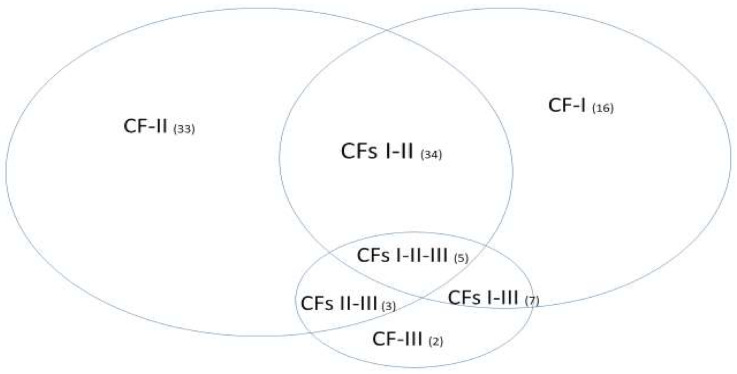

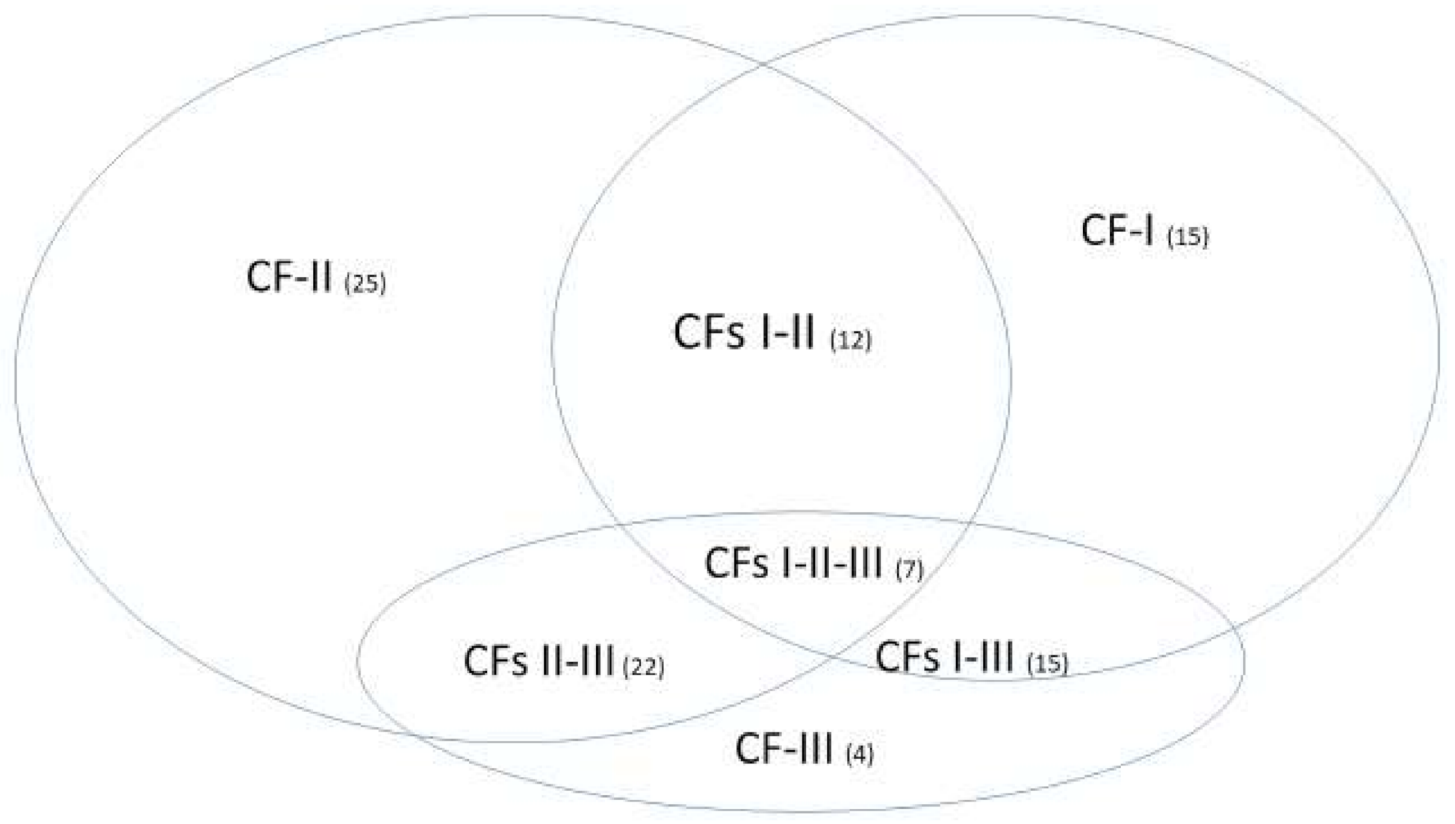
Venn diagrams of COVID-19 subtypes. The top panel is based on TPM data, and the bottom panel is based on expected counts data. The legend CF-I corresponds to the classifier formed by (KIAA1614, MND1, and SMG1); the legend CF-II corresponds to the classifier formed by (ABCB6, MND1, SMG1); the legend CF-III corresponds to the classifier formed by RIPK3; the legend CFs I-II corresponds to the combined classifier formed by (ABCB6, KIAA1614, MND1, SMG1); the legend CFs I-III corresponds to the combined classifier formed by (KIAA1614, MND1, RIPK3, SMG1); the legend CFs II-III corresponds to the combined classifier formed by (ABCB6, MND1, RIPK3, SMG1); the legend CFs I-II-III corresponds to the classifiers formed by all five genes. The numbers in the parentheses are the numbers of COVID-19 positive patients who are classified into the specific groups by the component classifiers or combined classifiers, respectively.

Figure 3 uses Venn diagrams to plot seven classified subtypes of 100 COVID-19 patients. In the figure, I-II means both CF-I and CF-II lead to the correct classification. All other combinations are interpreted similarly.

We first state that the more intersections of subareas, the more complicated the disease in a Venn diagram. For example, in the lower panel of the figure, we see that seven patients satisfy the classification conditions of all three competing classifiers. It turns out all of these seven patients are ICU COVID-19 patients. Using the lower panel as an example, we identify the ICU patients have the distribution CF-I (6), CF-II (8), CF-III (2), CFs-I-II (6), CFs-I-III (12), CFs-II-III (9), and CFs-I-II-III (7) and find that the disease severity (ICU) is positively correlated with the number of classifiers used. Based on this observation, we can conclude vaccines can benefit patients even if a particular type of vaccine only works for one signature pattern related to COVID-19 subtypes. On the other hand, if one particular type of vaccine can protect a particular COVID-19 subtype (or SARS-CoV-2 virus), this vaccine may not be effective for other COVID-19 subtypes (or SARS-CoV-2 viruses.) As a result, a fully vaccinated individual still has the risk of being infected. Furthermore, a recovered individual from an infected COVID-19 illness can get breakthrough infections again with other COVID-19 subtypes. Two recent papers report concerns about infection-enhancing anti-SARS-CoV-2 antibodies based on lab experiments [17,18]. This phenomenon may be explained by our new findings due to the existence of three genomic signature patterns and seven subtypes of COVID-19. Taking the SMG1 gene as an example, an increase (or a decrease) of SMG1 expression levels that are good for one signature pattern of COVID-19 will be bad for another signature pattern.

Note that the top and lower panels classify some patients into different subtypes. This phenomenon can be explained. In Section 3.4, we calculated the linear correlation coefficients between TPM and EC for each gene and saw there are nonlinear relationships, which lead to different classifications but are still accurate. Given that both TPM and EC lead to accurate results, it is safe to say that the identified five genes can be truly critical in studying COVID-19.

The identified subtype information opens a new research direction, new drug developments, and new refined personalized therapies. For example, in the diagnosis stage, medical doctors can use the final model to predict their patient’s COVID-19 status by calculating the risk and determining which of those seven groups the patient belongs to. In the treatment stage, those signature patterns can be used to study the effectiveness of drugs and treatments, i.e., conduct clinical trials based on classified groups. In the drug development stage, pharmaceutical companies can use the findings of critical genes to study new drugs. Finally, it can be hoped that mRNA-based therapies can be introduced using the critical genes’ information in the therapy stage.

### 3.6. A Conceptual Framework

From Section 3.3, we see that COVID-19 patients have higher combination expression values and COVID-19 free patients have lower combination expression values. With 100% sensitivity and 100% specificity, the competing classifiers derived in Section 3.3 build a biological equivalence to COVID-19 status. Equations (5) and (6) together with 100% sensitivity and 100% specificity reveal the hyperplanes formed by five genes, and their derived classifiers partition a five-dimension space into two subspaces (COVID-19 and COVID-19 free) in which there is a mathematical equivalence between COVID-19 and COVID-19 free. Here, the logic is from the fact of 100% sensitivity and 100% specificity, i.e., if A implies B, then not B implies not A, and if not A implies not B, then B implies A.

**Figure 4:**
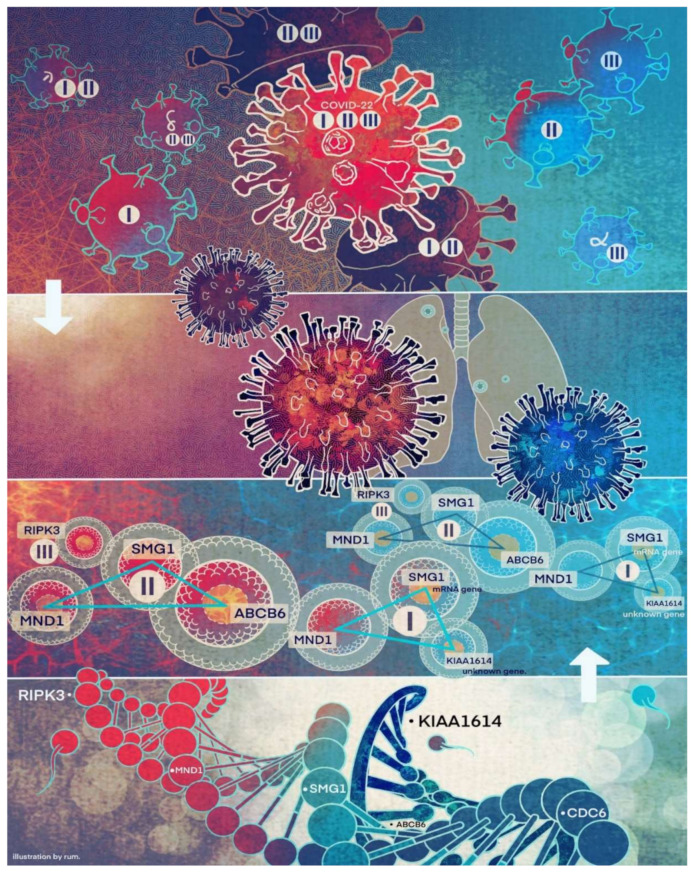
COVID-19 formation: a conceptual visualization of gene-gene relationship. Legend I stands for the signature formed by KIAA1614, MND1, and SMG1, legend II stands for the signature formed by ABCB6, MND1, and SMG1, and legend III stands for the signature formed by RIPK3. The author designed the concept and flowchart. Jing Zhang drew the figure.

In Section 4.1, we will use hydraulic engineering of a reservoir dam with cracks to describe COVID-19 variants metaphorically. Figure 4 uses the five genes identified in Section 3.3 and one additional gene, CDC6 (to be discussed in Section 4), to describe a conceptual framework for COVID-19 disease and variants formation dynamics.

In Figure 4, there are four layers. The first (top) layer stands for SARS-CoV-2 viruses entering a human’s interior body. The second layer shows the lung will be affected. The third layer describes gene-gene interaction signature patterns of COVID-19. The bottom layer is a conceptual illustration of a human genome sequence with the five critical genes placed on the sequence. In the figure, we use triangles to represent signature patterns (competing factor classifiers), with the genes on the nodes and the classifier number inside the center. RIPK3 is on its own as an absorbed triangle. There are two arrows to indicate the cause dynamics. With the triangles or RIPK3, the larger the triangle or the shape of RIPK3, the higher the severity of the COVID-19. Our conceptual idea is that after being infected with SARS-CoV-2 (top-down direction), the virus triggers the signature patterns to function, i.e., making the triangles (the shape) larger. However, simultaneously, the human’s immune system starts to function (bottom-up direction), and the vaccine also starts to function; therefore, the areas of triangles (shape) can be reduced, or the edges of the triangles can be broken, i.e., two ways of fighting against the virus. Depending on which direction (infection or ‘killing’ the virus) is more effective, an infected individual may be fully recovered from COVID-19 disease or suffer much more severe COVID-19 symptoms.

On the other hand, the genomic signature patterns of a COVID-19 patient represent the advanced (deep level) gene-gene interactions. A change in the size of the triangle may trigger new variants to form and transmit to other individuals, i.e., an analog to the hydraulic engineering example in Section 4.1.

### 3.7. Clinic Characteristics

In this section, we present the distributions of clinic variables, sex, age, and ICU status, and seven subtypes, in Table 5.

Table 5 shows that ranges of age among all subtypes are similar, i.e., from 20 s to 80 s. More males were hospitalized than females. Patients with the subtype sub-I were more likely (6:9 vs. 8:17) to need ICU than those with the subtype sub-II. The reason why this could happen is not known. Comparing CF-I and CF-II, we see that KIAA1614 is an unknown gene, and the sign of SMG1 is negative in CF-I, which may be the causal factor. In addition, sub-V and sub-VI share similar features, and sub-V contains KIAA1614 and a negative coefficient sign of SMG1. We note that if all things are wrong, i.e., the sub-VII subtype, all patients were ICU patients. Therefore, it tells us the sub-I subtype is more puzzling, likely due to the unknown gene KIAA1614.

### 3.8. Analysis of the Third Dataset

Table 6 reports the fitted coefficient values for four critical genes and related sensitivities and specificities of competing risk classifiers using raw counts.

Comparing Table 1 and Table 6, we can immediately see that Table 6 does not contain a CF-III classifier (RIPK3), and the coefficient signs of KIAA1614 in CF-I and SMG1 in CF-II are negative, which are different from their counterparts in Table 1. This observation justifies that there are more than three genomic signature patterns and seven subtypes. Recall that the controls in the first and second datasets were hospitalized patients with non-COVID-19 diseases, while the control in this third dataset is healthy subjects. As a result, it is safe to conclude that the five genes, ABCB6, KIAA1614, MND1, RIPK3, and SMG1, are truly COVID-19 specific, and the newly proposed classification method is a powerful tool.

Note that the coefficients of MND1 in both Table 1 and Table 6 are uniformly positive. This observation suggests that MND1 (meiotic nuclear divisions 1) may be the most important gene related to COVID-19.

Table 7 lists gene expression values, competing classifiers’ values among all patients in the third dataset.

From Table 7, we can see that MND1 expression values show significant difference between the healthy group and the COVID-19 group.

## 4. Conclusions and Discussions

### 4.1. Discussions

The proposed method is different from the current diagnostic methods in several ways. First, our new method (S4) theoretically leads to finding the smallest number of genes with clear signature patterns which are interpretable. Second, our method directly deals with heterogeneous populations and performs natural clustering and classifications of samples into their respective groups. Third, our proposed method can describe gene-gene interactions and gene-subtypes interactions.

Simultaneously observing the same set of five genes for two different datasets has not been reported in published literature. In our opinion, those published genes by many other studies are more like at the surface level (biologically directly related to the disease) based on the analysis methods used, and the new set of five genes in this work is at the deep level or the root level, where genes are not directly related to the diseases by the present biological knowledge. Furthermore, many reported key genes are based on their individualized expression value changes and significance, i.e., not based on gene-gene interactions. As a result, treatments are palliative, and the disease is difficult to cure. The findings in our new research are based on nonlinear and competing risk factors interactions, which is an advanced gene-gene interaction mechanism. Our proposition is that the biomedical discovery of new variants of COVID-19 is only the surface level of the virus (diseases). More profound, underlying “competing factors” of the virus need to be studied. Metaphorically, an expert in hydraulic engineering finds a dam with cracks and treats them on the surface. However, the reservoir has an interconnected water dynamic below the surface that will further impact other points of the dam. As a result, it will cause new cracks unless there is a fundamental treatment solution with the entire structure in mind. Similarly, scientists may observe the variants (rather passively) and develop vaccines in response to the variants. However, knowledge of the virus’s deeper advanced genomic architecture that will systematically cause other mutations is limited. Traditional methods in statistics, machine learning, and AI are limited to understanding COVID-19 from surface-level observations. However, our innovative method has achieved significant results in identifying and understanding COVID-19 genomic signatures.

The newly identified genes and their combinations may be used as new biomarkers. In our opinion, traditional methods (e.g., PCR, serology) are directly associated with the disease symptoms, i.e., they do not provide pathological characteristics; they are a onefold indicator. On the other hand, the new classifier is a multifold indicator that can further divide the disease into subgroups (variants may be another word). In addition, the new classifier shows gene-gene interactions and advanced (or root) structures.

This work has verified that when all component classifiers simultaneously classify a group of patients as COVID-19-positive, these patients are ICU patients (Section 3.4), which definitely points out the advanced genomic structure of COVID-19 disease.

In the literature and the current practice, tremendous efforts have been made to study COVID-19 genomic sequences, variants and their impacts, and vaccine effectiveness. However, the progress on the pathological causes of COVID-19 and the functional effects of genes is still limited. In terms of computational medicine, our new work is the first to accurately define the functional effects of five critical genes and lead to the mathematical and biological equivalence between five genes and COVID-19 status. Furthermore, this paper introduces an advanced machine learning algorithm that identifies five essential genes, which further determine three genomic signature patterns and seven subtypes of COVID-19 with high accuracy. The final classifiers are expressed by explicit mathematical equations which are interpretable and can guide medical practice. In addition, new graphical diagnostic tools are introduced. Besides the striking advance in studying genomic signature patterns of COVID-19, our work also sheds new light on computational medicine, genetic studies, informatics, algorithm and machine learning, and statistics.

We realized readers would ask about the model overfitting and robustness. Please note that our model is fitted to three different datasets and has reached the highest accuracy. Each dataset has its heterogeneous patterns (subgroups). Datasets are measured at different scales. It is hard for the existing models to simultaneously fit such datasets and get satisfactory accuracy, not to mention the interpretability of the fitted models. Using three such datasets naturally serves as cross-validation and robust checking. It turns out our new approach is robust.

In many scenarios, a 100% accuracy may be thought of as “too good to be true”. However, “too good to be true” may also be dangerous to use to guide science discovery and innovation. In many applied sciences, the truths can be simple but not straightforward. Complicating or aggravating the problem can mask the nature of the problem. Blindly insisting on “too good to be true” may miss ample opportunities of finding the truth. In contrast, we know it is hard to see the forest through the trees.

One may argue that the dataset we used in this analysis is not large enough as it has only 126 samples with 19,472 predictors in the first two datasets and 34 samples with 64,083 genes in the third dataset. It is, of course, preferable to have a large dataset. Nevertheless, we argue that the conclusions and inferences are trustworthy with a convincing accuracy on three different datasets that show nonlinear and heterogeneous relationships.

On the other hand, if an approach cannot gain a satisfactory performance with a small dataset, applying it to a large dataset can be a wrong strategy as it may lead to wrong or suboptimal conclusions.

A natural question is whether or not the high accuracy is by chance. Note that each of our component competing classifiers has reached 100% specificity, which may be true with a probability smaller than $1/2^{26}\;=\;1.0\;\times \;10^{-8}$ by chance. In addition, when all three signature patterns are satisfied, all classified patients are lab-confirmed ICU patients, which indicates it cannot be by chance.

We have used analogies to interpret our modeling strategy. We note that our proposed method is still in its primary stage of development, just started in 2021. Researchers are still not very familiar with the method. Once the proposed method becomes a standard method, the introduction of the method and analogies will no longer be needed.

### 4.2. Conclusions

As discussed in Section 1 and Section 3, the three signature patterns and seven subtypes maintain the most important biological informatics of COVID-19. This set of genes is the only set that leads to accurately classifying hospitalized COVID-19 patients, including ICU patients, into their respective groups. Unless a different new discovery of other advanced structures of genes other than these five genes can be obtained, and such a new discovery (if exists) can fully explain the three signature patterns and seven subtypes discussed in this paper, these five critical genes and their derived three signature patterns and seven subtypes remain the most informative findings.

When a model is fitted to the whole dataset and leads to (nearly) perfect accuracy, it will uniformly work for partitioned data as long as the partition is balanced to all heterogeneous subgroups. This is the case in our analysis. On the other hand, we have not seen any published papers that used the “standard” procedure to lead to accurate prediction.

We note that multiplying a constant to Equations (5) and (6) will not change the classification results and the shapes in Figure 1 and Figure 2 with a convincing accuracy being achieved. However, the color strengths will be changed. Such a phenomenon justifies the use of signatures to describe the advanced gene-gene interaction structures. Using this idea, we can express (6) into the following equivalent forms:

(11) $$\begin{array}{l} \mathrm{CF}-I\;(\mathrm{EC}):-0.3303\;+\;3.4153\;\times \;\frac{\mathrm{KIAA}1614}{231.9296}-0.1248\;\times \;\frac{\mathrm{SMG}1}{370.2751}\;+\;0.2177\;\times \;\frac{\mathrm{MND}1}{28.6399}\\ \mathrm{CF}-\mathrm{II}\;(\mathrm{EC}):-0.7378-0.4620\;\times \;\frac{\mathrm{ABCB}6}{71.6741}\;+\;0.0654\;\times \;\frac{\mathrm{SMG}1}{305.4532}\;+\;0.9093\;\times \;\frac{\mathrm{MND}1}{26.9660}\\ \mathrm{CF}-\mathrm{III}\;(\mathrm{EC}):\;6.9283-0.3921\;\times \;\frac{\mathrm{RIPK}3}{42.5580}\\ \mathrm{CF}(\mathrm{EC})\;=\;\max\{\mathrm{CF}-I(\mathrm{EC}),\;\mathrm{CF}-\mathrm{II}(\mathrm{EC}),\;\mathrm{CF}-\mathrm{III}(\mathrm{EC})\} \end{array}$$

Equation (11), after applying scale changes, shares similar signatures as in Equation (5). Considering the nonlinear relationships between TPM data and EC data, this observation again proves our proposed competing factor classifiers are robust.

Our findings can be used to develop precision test kits for testing COVID-19 and to evaluate the function and performance of already implemented vaccines, i.e., used as new antibody indexes. Interpretable and implementable formulas are given in the paper. After a COVID-19 case is confirmed, personalized treatments can be implemented. For example, increasing or decreasing levels of critical genes based on the identified COVID-19 subtype can be crucial to the patients’ recovery. Using the relationship determined in the findings, antiviral drugs can be developed. Mathematically, the new objective function of Equation (4) is a mixture of combinatorial optimization and continuous optimization. It is a new type of classification benchmark. It is expected that this new classification formula will motivate research in statistics, computational mathematics, computer science, and many applied sciences. The findings can motivate many new types of research in COVID-19 studies and other studies, e.g., cancer studies. Many finished studies can re-start new investigations using the new methodology.

### 4.3. Future Perspectives

With the mathematical equivalence between Equations (6) and (11), different numbers of patients in different subtypes and in the control will not change the classification results. With the specificities of each individual classifier (CF-i) reaching 100%, the signature patterns (not presented in Section 3.3) of patients in the control will be the same, i.e., just one type. This phenomenon shows that the identified five critical genes are COVID-19 specific. One can apply our method to study critical genes of disease types (also other respiratory diseases) in the control, i.e., assign the related samples in the treatment group and specify some other types of diseases or normal cases as the control.

We want to hypothesize that the discovered COVID-19 variants (alpha, beta, delta, lambda, mu, omicron, etc.) may be connected to different signature strengths in our discovered signature patterns. COVID-22 in Figure 4 means that SARS-CoV-2 variants in 2022 can be combinations of several subtypes (variants), i.e., they are no longer the same types as in 2019. Mathematically, hyperplanes in geometry formed by Equations (5) and (11) contain a subspace that can be further partitioned into subspaces. Therefore, we hypothesize that these variants may fall into separable subspaces. After obtaining new data with variants information, this hypothesis can be tested, or additional genes may be involved. For example, in the breast cancer study mentioned earlier, triple-negative breast cancer was accurately separated from other types of breast cancer using three genes identified by the S4 classifier. Furthermore, the discovered genomic signature patterns and COVID-19 subtypes are intrinsic no matter what variants have been identified or will be identified. Given our proven mathematical and biological equivalences, if these innate signature patterns and subtypes cannot be treated and fully studied now, they will cause trouble in the future again. In addition, with available data related to various variants, our study approach may be able to reveal the causes of higher transmission or mortality of specific variants.

Using classifiers CF-I, -II, and -III as new biomarkers, we can study other potential genes that are highly correlated with these biomarkers. For example, based on the first and second datasets, the most highly correlated five genes to each new biomarker lead to a total of fifteen genes: DBN1, LY6G6C, TMEM54, MTMR1, SNORC, ANP32E, ATAD2, SMC2, ZWILCH, SMC4, C6orf47, STRADA, LRSAM1, UNC93B1, and SASH3, which were listed in the Introduction.

Finally, in our analyses, we also found the gene CDC6 (cell division cycle 6) can be informative. Its combination with ZNF282 (zinc finger protein 282) can lead to 97.62% accuracy (98% sensitivity, 96.15% specificity), and its combination with both ZNF282 and CEP72 (centrosomal protein 72) can lead to 98.41% accuracy (99% sensitivity, 96.15% specificity). We found the high expression level of CDC6 increases risk while the high expression levels of ZNF282 and CEP72 decrease risk. Although they did not lead to the best accuracy as those five genes identified in Section 3 did, such a performance is already better than other published gene combinations. From the literature, the gene CDC6 is a protein essential for the initiation of DNA replication, while ZNF282 is known to bind U5RE (U5 repressive element) of HLTV-1 (human T cell leukemia virus type 1) with a repressive effect, but little is known of its expression and function otherwise. The gene CEP72 coded protein is localized to the centrosome, a non-membraneous organelle that functions as animal cells’ major microtubule-organizing center. Zhang (2022) hypothesizes that CDC6 is a protein essential for the initiation of RNA replication of COVID-19 [19].

### 4.4. Limitation of the Study

Data used in this study are from hospitalized patients’ blood samples. We are not sure whether or not the identified genes work for data sampled from those non-hospitalized COVID-19 patients or asymptomatic patients. Solving optimization problems (4) involves combinatorial optimization, integer programming, and continuous programming. The computational complexity is extremely high, and we have not figured out how to define the complexity. We used an extensive Monte Carlo search method to find the best solution. However, we cannot be sure whether additional sets of genes can also be the optimal solutions even if our finding of five genes is already the best (smallest) subset of genes with the desired accuracy in the study of COVID-19. Although we have established the mathematical and biological equivalences, we cannot tell our findings are the causes or the results. Although we have identified functional effects via gene-gene interactions and gene-subtype interactions of the five genes, we have not identified how genes interact with each other and their causal directions. We are working in this direction. As the proposed method is still at the early stage of development, just started in 2021, assumptions for future use of the results of this study have been applied. Finally, due to the lack of available new blood sampled data for new variants, it is difficult to infer the risks of variants. Furthermore, what we have obtained are computational results and it is not necessary the case that the results will follow the same pattern in vitro/in vivo or in clinics.

## Figures and Tables

**Table 1:** Performance of individual classifiers and combined max-competing classifiers in Equations (5) and (6).

Classifiers	Intercept	ABCB6	KIAA1614	MND1	RIPK3	SMG1	Accuracy %	Sensitivity %	Specificity %
CF-I (TPM)	−0.3303		3.4153	0.2177		−0.1248	69.84	62	100
CF-II (TPM)	−0.7378	−0.462		0.9093		0.0654	80.16	75	100
CF-III (TPM)	6.9282				−0.3921		34.13	17	100
CFmax							100	100	100
CF-I (EC)	−0.7877		0.0351	0.0181		−0.0008	59.52	49	100
CF-II (EC)	−4.6701	−0.0408		0.2134		0.0014	73.02	66	100
CF-III (EC)	3.1584				−0.0042		58.73	48	100
CFmax							100	100	100

**Table 2:** TPM data: Expression values of the five critical genes, competing classifier factors, and predicted probabilities.

ID	ABCB6	KIAA1614	MND1	RIPK3	SMG1	CF-I	CF-II	CF-III	CF(TPM)	P-I	P-II	P-III	P-(TPM)
C1	2.77	0.78	0.6	39.22	30.2	−1.30	0.50	−8.45	0.50	0.21	0.62	0.00	0.62
C2	2.52	0.39	2.91	26.82	20.33	−0.90	2.07	−3.59	2.07	0.29	0.89	0.03	0.89
C3	3.14	1.07	1.26	40.13	16.16	1.58	0.01	−8.81	1.58	0.83	0.50	0.00	0.83
C4	2.11	0.59	1.99	25.49	23.33	−0.79	1.62	−3.07	1.62	0.31	0.84	0.04	0.84
C5	1.14	0.43	0.75	23.62	20.4	−1.24	0.75	−2.33	0.75	0.22	0.68	0.09	0.68
…													
C103	3.37	0.54	0.7	27.31	5.43	0.99	−1.30	−3.78	0.99	0.73	0.21	0.02	0.73
NC1	2.17	0.15	0.16	18.89	3.57	−0.23	−1.36	−0.48	−0.23	0.44	0.20	0.38	0.44
NC2	1.83	0.25	0.25	25.77	8.24	−0.45	−0.82	−3.18	−0.45	0.39	0.31	0.04	0.39
…													
NC26	1.36	0.06	1.36	19.9	1.47	−0.01	−0.03	−0.87	−0.01	0.50	0.49	0.29	0.50

C1,……, C103 are hospitalized COVID-19 patient IDs, and NC1,……, NC26 are COVID-19 free patient (also hospitalized) IDs. CF and P are definded in Equations (5)–(10).

**Table 3:** EC data: Expression values of the five critical genes, competing classifier factors, and predicted probabilities.

ID	ABCB6	KIAA1614	MND1	RIPK3	SMG1	CF-I	CF-II	CF-III	CF(TPM)	P-I	P-II	P-III	P-(TPM)
C01	143	141.2	8	1217	8952	−2.88	3.33	−0.47	3.32678	0.05	0.97	0.39	0.97
C02	82	46.13	25	539	3913	−1.86	2.62	0.213	2.62006	0.13	0.93	0.55	0.93
C03	159	195.76	17	1250	4803	2.53	−1.02	−0.5	2.53444	0.93	0.26	0.38	0.93
C04	92	92.4	23	685	6001	−1.95	4.61	0.067	4.61279	0.12	0.99	0.52	0.99
C05	58	78.36	10	734	6085	−2.74	3.34	0.018	3.33954	0.06	0.97	0.50	0.97
…													
C103	290	169.66	16	1451	2773	3.23	−9.33	−0.7	3.23146	0.96	0.00	0.33	0.96
NC01	151	37.54	3	826	1479	−0.6	−8.19	−0.07	−0.074	0.35	0.00	0.48	0.48
NC02	140	67.34	5	1199	3688	−1.3	−4.32	−0.45	−0.447	0.21	0.01	0.39	0.39
…													
NC26	126	22	35	1168	814.2	−0.04	−1.24	−0.42	−0.03501	0.49	0.22	0.40	0.49

C1, ……, C103 are hospitalized COVID-19 patient IDs, and NC1, ……, NC26 are COVID-19 free patient (also hospitalized) IDs. CF and P are definded in Equations (5)–(10).

**Table 4:** Pairwise Pearson’s correlation coefficients: The upper triangle is for TPM data, and the lower triangle is for EC data.

	ABCB6	KIAA1614	MND1	RIPK3	SMG1
ABCB6	1	0.6931	0.3448	−0.1204	0.2138
KIAA1614	0.5209	1	0.1609	0.1688	0.5948
MND1	0.4009	0.1163	1	−0.1328	0.1276
RIPK3	−0.0727	−0.1608	−0.0769	1	0.2293
SMG1	−0.0955	0.4681	−0.0137	−0.1762	1

**Table 5:** Distributions of basic clinical and pathological characteristics.

Subtypes	Number of Patients	Age (Years)		Sex		ICU	
		Median	Range	Male	Female	Yes	No
sub-I	15	73	33–85	12	3	6	9
sub-II	25	58	27–86	15	10	8	17
sub-III	4	49.5	29–87	3	1	2	2
sub-IV	12	57.5	24–81	3	9	6	6
sub-V	15	68	41–83	9	6	12	3
sub-VI	21	55	35–90	16	6	9	13
sub-VII	7	50	21–71	4	3	7	0

**Table 6:** Performance of individual classifiers and combined max-competing classifiers.

Classifiers	Intercept	ABCB6	KIAA1614	MND1	RIPK3	SMG1	Accuracy %	Sensitivity %	Specificity %
CF-I (Raw)	9.0357		−0.0611	0.1628		−0.0089	97.06	94.12	100
CF-II (Raw)	9.2613	−0.2191		0.1963		–0.0081	97.06	94.12	100
CFmax							100	100	100

**Table 7:** The third data: Expression values of four critical genes, their classifier values, risk probabilities.

ID_REF	Severity	ABCB6	KIAA1614	MND1	SMG1	CF-I	CF-II	CFmax	P-I	P-II	P-Max
GSM4614985	Convalescent	1	7	2	1107	−0.92	0.47	0.47	0.29	0.61	0.61
GSM4614986	Moderate	12	8	23	975	3.61	3.25	3.61	0.97	0.96	0.97
GSM4614987	Severe	2	12	4	731	2.45	3.69	3.69	0.92	0.98	0.98
GSM4614988	Severe	13	5	48	1033	7.35	7.47	7.47	1.00	1.00	1.00
GSM4614989	ICU	9	5	10	655	4.53	3.95	4.53	0.99	0.98	0.99
GSM4614990	Severe	9	6	11	1010	1.47	1.27	1.47	0.81	0.78	0.81
GSM4614991	Moderate	11	11	10	646	4.24	3.58	4.24	0.99	0.97	0.99
GSM4614992	Severe	4	6	10	733	3.77	4.41	4.41	0.98	0.99	0.99
GSM4614993	ICU	17	13	9	503	5.23	3.23	5.23	0.99	0.96	0.99
GSM4614994	Severe	4	7	12	774	3.67	4.47	4.47	0.98	0.99	0.99
GSM4614995	Moderate	9	8	23	1232	1.33	1.83	1.83	0.79	0.86	0.86
GSM4614996	ICU	4	15	7	752	2.57	3.67	3.67	0.93	0.98	0.98
GSM4614997	Severe	14	10	27	894	4.86	4.25	4.86	0.99	0.99	0.99
GSM4614998	ICU	7	14	21	965	3.01	4.03	4.03	0.95	0.98	0.98
GSM4614999	Severe	12	12	23	1233	1.07	1.16	1.16	0.75	0.76	0.76
GSM4615000	Severe	11	8	5	1039	0.11	−0.58	0.11	0.53	0.36	0.53
GSM4615001	Moderate	12	6	16	953	2.79	2.05	2.79	0.94	0.89	0.94
GSM4615003	Healthy	7	4	1	1157	−1.34	−1.45	−1.34	0.21	0.19	0.21
GSM4615006	Healthy	5	10	1	1040	−0.67	−0.06	−0.06	0.34	0.48	0.48
GSM4615008	Healthy	9	6	1	1354	−3.22	−3.48	−3.22	0.04	0.03	0.04
GSM4615011	Healthy	5	10	0	1285	−3.01	−2.24	−2.24	0.05	0.10	0.10
GSM4615014	Healthy	16	6	1	1641	−5.77	−7.34	−5.77	0.00	0.00	0.00
GSM4615016	Healthy	5	2	3	1250	−1.72	−1.37	−1.37	0.15	0.20	0.20
GSM4615019	Healthy	11	15	8	1164	−0.94	−1.01	−0.94	0.28	0.27	0.28
GSM4615022	Healthy	11	8	2	1246	−2.22	−2.85	−2.22	0.10	0.05	0.10
GSM4615025	Healthy	13	25	2	1220	−3.02	−3.08	−3.02	0.05	0.04	0.05
GSM4615027	Healthy	7	5	3	1327	−2.59	−2.43	−2.43	0.07	0.08	0.08
GSM4615030	Healthy	9	6	2	1138	−1.13	−1.54	−1.13	0.24	0.18	0.24
GSM4615032	Healthy	8	8	0	1264	−2.70	−2.73	−2.70	0.06	0.06	0.06
GSM4615033	Healthy	7	2	1	1201	−1.61	−1.80	−1.61	0.17	0.14	0.17
GSM4615034	Healthy	8	5	5	1659	−5.22	−4.95	−4.95	0.01	0.01	0.01
GSM4615035	Healthy	12	12	0	1371	−3.90	−4.47	−3.90	0.02	0.01	0.02
GSM4615036	Healthy	10	7	5	1337	−2.48	−2.78	−2.48	0.08	0.06	0.08
GSM4615037	Healthy	6	8	1	1461	−4.29	−3.69	−3.69	0.01	0.02	0.02

## Data Availability

The datasets are publicly available. The data links are stated in the Data Description Section.

## Supplementary Materials

The following supporting information can be downloaded at: https://www.mdpi.com/article/10.3390/vaccines10050761/s1, Tables S1 and S2 with complete patients’ IDs and expression values corresponding to Table 2 and Table 3 in the main text. Real data and computer outputs are in a supplementary file available online and submitted together with this paper. In addition, a MATLAB^®^ demo code for solving a final dataset example in Equation (4) (λ_2_ = 0) is also available.
